# Diurnal Habitat Selection and Use of Wintering Bar-Headed Geese (*Anser indicus*) Across Heterogeneous Landscapes on the Yunnan–Guizhou Plateau, Southwest China

**DOI:** 10.3390/ani15192826

**Published:** 2025-09-28

**Authors:** Chao Li, Hong Liu, Ziwen Meng, Weike Yan, Linna Xiao, Yu Lei, Xuyan Zhao, Zhiming Chen, Qiang Liu

**Affiliations:** 1 College of Ecology and Environment (College of Wetlands), Southwest Forestry University, Kunming 650224, China; chaoli20010611@163.com (C.L.); liuhong19990528@163.com (H.L.); ziwen_meng@126.com (Z.M.); ywk445027709@163.com (W.Y.); linnaxiao@mail.bnu.edu.cn (L.X.); leiallen521@126.com (Y.L.); 2 Napahai Provincial Nature Reserve Administration Bureau, Shangri-La 674499, China; 18287864941@163.com (X.Z.); leileiren1@163.com (Z.C.); 3 Yunnan Key Laboratory of Plateau Wetland Conservation, Restoration and Ecological Services, Kunming 650224, China

**Keywords:** *Anser indicus*, habitat selection, habitat use, random forest, satellite tracking

## Abstract

Wetland loss and human activities are forcing migratory waterbirds to rely on alternative habitats such as croplands, yet their adaptive habitat use across contrasting landscape contexts remains unclear. The Bar-headed Goose (*Anser indicus*) is a key indicator species in the wetland ecosystems of the Yunnan–Guizhou Plateau. Comparing differences in its wintering habitat selection and utilization is of great significance for understanding its ecological adaptation mechanisms and formulating regional wetland conservation strategies. In this study, we compared the diurnal habitat use during the wintering period of Bar-headed Geese at three wetlands (Nianhu, Caohai, and Napahai) representing distinct landscape contexts. We used GPS satellite tracking and dynamic Brownian bridge movement modeling, combined with random forest analysis of environmental variables, to quantify diurnal habitat use and selection at each site. Our results revealed significant regional differences in habitat use. In the agriculture-dominated wetlands (Nianhu and Caohai), geese primarily utilized cropland and marsh habitats (Nianhu: cropland 45.88% ± 30.70%, marsh 42.55% ± 33.17%; Caohai: cropland 62.33% ± 12.16%, marsh 28.61% ± 13.62%). In contrast, at Napahai, which is dominated by natural habitats, geese primarily used grassland (65.92% ± 20.01%) and marsh (26.85% ± 21.88%), with minimal use of cropland (4.21% ± 7.00%). Diurnal habitat selection was influenced by multiple environmental factors, with distinct regional differences identified through random forest modeling. In Nianhu, key factors included distance to supplemental feeding site, distance to grassland, distance to woodland, and distance to open water. In Caohai, distance to grassland, distance to nocturnal roost site, distance to settlement, and distance to open water were significant drivers. In Napahai, distance to nocturnal roost site, distance to open water, and distance to marsh were the most influential (all with *p* < 0.01), reflecting flexible behavioral responses. Based on these findings, we recommend region-specific conservation management strategies. Specifically, supplemental feeding at Nianhu should be strictly regulated. Agricultural planning in farming areas should account for the habitat needs of wintering waterbirds. Grassland and marsh habitats at Napahai should also be more effectively protected.

## Simple Summary

This study investigated differences in diurnal habitat selection and use by wintering Bar-headed Geese across wetlands with varying landscape characteristics. Using satellite tracking data and habitat modeling, we found that the Bar-headed Goose exhibited distinct diurnal habitat use patterns among different wintering wetlands. In the agriculturally dominated wetlands of Nianhu and Caohai, geese primarily utilized croplands and marshes, while in the more natural Napahai wetland, they primarily utilized natural grasslands and marshes. These behavioral differences highlight the geese’s behavioral flexibility in adjusting to diverse landscape conditions. Our findings provide a scientific basis for developing region-specific wetland bird conservation strategies. We emphasize the need for management actions tailored to local landscape characteristics. Such actions include carefully managing supplemental feeding, preserving cropland–wetland mosaics, and optimizing water-level management to effectively conserve wintering waterbirds on plateau wetlands.

## 1. Introduction

Global biodiversity loss, largely driven by habitat destruction and fragmentation, is reshaping Earth’s ecosystems at an unprecedented rate [1,2,3]. Migratory waterbird populations have generally declined in recent years, with agricultural intensification, wetland reclamation, and climate change identified as major contributors [4,5,6,7]. Wetland degradation forces many migratory waterbirds to increasingly rely on alternative habitats, such as croplands, grasslands, and reservoirs, to replenish their energy reserves. However, resources in these substitute habitats are often unstable and accompanied by frequent human disturbances [8,9,10]. Understanding how migratory birds balance resource acquisition and disturbance risk through habitat selection is therefore crucial. This not only reveals the plasticity of avian behavior [11,12,13] but also provides an important means of assessing wetland ecological functions and evaluating the impacts of human activities [14,15,16].

The Bar-headed Goose (*Anser indicus*) is a flagship migratory waterbird and a key indicator species along the Central Asian Flyway. It is well-known for its unique Himalaya-crossing migration route and specialized physiological adaptations to high-altitude environments [17,18,19]. This species breeds primarily on the Qinghai–Tibetan Plateau and adjacent high-altitude wetlands, wintering in regions such as the Yunnan–Guizhou Plateau and the Indian subcontinent. Its population dynamics are closely linked to habitat quality across these regions [20,21,22,23]. The wintering period represents the stage of the avian life cycle characterized by the greatest ecological stress. During this critical time, individuals face extreme weather conditions, fluctuations in resource availability, and frequent human activities. Habitat selection decisions made during this period directly influence energy replenishment efficiency and overall population viability [13,24]. Previous research has revealed the habitat-use characteristics of Bar-headed Geese at migratory stopover sites [25] and local wintering areas [26]. However, these studies have typically been limited to individual locations, lacking systematic comparisons across contrasting landscape contexts with differing human land-use. Consequently, the factors shaping habitat selection and use across natural versus agriculture-dominated wetlands remain insufficiently understood.

The diverse wetland landscapes of the Yunnan–Guizhou Plateau encompass distinct human-use regimes [27], offering an exceptional opportunity to study migratory waterbird behavior within heterogeneous habitats. As a core wintering region for Bar-headed Geese along the Central Asian Flyway, this area’s distinctive mosaic landscape—combining croplands and wetlands—provides essential food resources (e.g., residual crops in agricultural fields) and secure refuges (e.g., shallow marsh habitats). Bar-headed Geese are the dominant wintering waterbird species in agricultural zones on the plateau, preferentially utilizing mosaic landscapes where croplands and wetlands coexist. Consequently, these populations may be more vulnerable to human activities compared with species that inhabit purely natural environments [26,28]. Earlier monitoring has indicated significant differences in habitat use among Bar-headed Goose populations across the region’s three major wetlands: the populations at Nianhu and Caohai are heavily reliant on agricultural landscapes, whereas the Napahai population primarily utilizes marsh-grassland ecotones. Given this background, we hypothesized that Bar-headed Geese exhibit adaptive habitat selection patterns across heterogeneous wetlands, selecting habitats according to local resource availability. Considering the ecological requirements of the geese and the unique local conditions, we quantified distance-based environmental factors related to nocturnal roost sites, foraging areas, and human-activity covariates. Through this approach, we aimed to explore how wintering Bar-headed Geese adjust their habitat selection across different landscape structures and how these adjustments ultimately shape distinct habitat-use patterns.

In this study, we selected Nianhu, Caohai, and Napahai as representative wintering wetlands and employed an integrative approach combining satellite tracking, remote sensing classification, dynamic Brownian bridge movement modeling, and random forest analysis. Our specific objectives were (1) to identify regional differences in diurnal habitat use of wintering Bar-headed Geese; (2) to quantify the relative influence of various environmental factors on diurnal habitat selection and compare these factors among regions; and (3) to provide a theoretical basis and empirical evidence for informing conservation management of wintering waterfowl in plateau wetlands, thereby promoting a shift from a generalized conservation approach toward regionally differentiated management strategies.

## 2. Materials and Methods

### 2.1. Study Area

**Figure 1:**
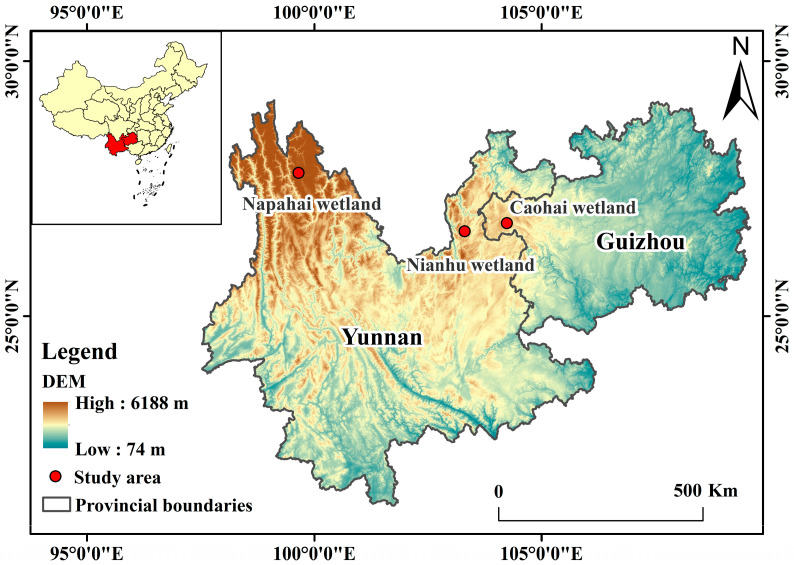
Regional context and locations of the three study wetlands on the Yunnan–Guizhou Plateau. Red circles mark Nianhu, Caohai, and Napahai; provincial boundaries are shown in gray. The background is a DEM (low–high). The inset map indicates the position of Yunnan and Guizhou within China. A north arrow, latitude–longitude graticule (WGS84), and a 0–500 km scale bar are provided for reference.

Nianhu Wetland (103°12′–103°22′ E, 26°38′–26°44′ N) lies within the Huize National Nature Reserve for Black—necked Cranes (*Grus nigricollis*) in Yunnan Province and comprises the Daqiao and Changhaizi sections (Figure 1). The Daqiao section is one of the principal wintering sites for Bar—headed Geese on the Yunnan–Guizhou Plateau, at elevations of 2470–3092 m. Nianhu has a cold, high-elevation montane climate, with a mean annual temperature of 9.6 °C and relatively high annual precipitation. On average, the area experiences about 40 days of snowfall, 50 days of snow cover, and 45 days of ice cover per year [29]. Dominant habitat types include open water, marsh, cropland, grassland, woodland, and settlements. Croplands are subject to intensive human activity, especially during the harvest (October–November) and planting (February–March) seasons [30]. Recent surveys indicate more than 4000 Bar-headed Geese winter in the reserve each year [31].

Caohai Wetland (104°10′–104°20′ E, 26°47′–26°52′ N) is located in the Wumeng Mountains of Weining County, Guizhou Province (Figure 1). It is a national nature reserve and the largest and most representative plateau freshwater lake wetland in the province. The open-water area covers approximately 25 km^2^, with a mean depth of less than 1.5 m. Average elevation is 2171.7 m, and basin elevation decreases gradually from the center northward. Caohai has a subtropical plateau monsoon climate, with a mean annual temperature of 10.9 °C and approximately 1000 mm of precipitation. Winters are dry, summers are wet, and the area receives an average of 1805.4 h of sunshine per year. Habitat types include open water, marsh, and grassland complexes [32]. Owing to its relatively intact ecological structure and function, Caohai is regarded as a representative subtropical plateau wetland ecosystem in China and serves as an important wintering and stopover site for migratory birds in Southwest China. Each winter, it supports over 80 waterbird species totaling over 80,000 individuals [33]. Among these, 2000–5000 Bar-headed Geese winter at Caohai each year, with a mean of approximately 3500 individuals [34].

Napahai Internationally Important Wetland (99°37′–99°45′ E, 27°48′–27°55′ N) is situated in Shangri-La City, Diqing Tibetan Autonomous Prefecture, Yunnan Province, and is protected as a provincial-level nature reserve (Figure 1). Napahai is one of China’s most representative seasonal alpine lake wetlands at low latitude and high elevation, and it was designated a Ramsar site in 2004. During the summer wet season, the lake expands, reaching water depths of 4–5 m; in the autumn–winter dry season, it contracts rapidly to a surface area of about 500 ha, exposing a marshy meadow that supports an alpine wetland ecosystem. The site has a mean annual temperature of 5.4 °C (extremes 25.1 °C and −25.4 °C) and receives approximately 620 mm of precipitation annually; most falls as snow from September through May. Mean relative humidity is 69.9%, and annual sunshine totals about 2190 h, with more than 200 h in each winter month [35]. Napahai is the most important wintering area for the central migratory population of the Black-necked Crane (an endangered Class I protected species in China) [36]. It also hosts approximately 4000 wintering Bar-headed Geese each year [37].

### 2.2. Data Collection

With approval from local forestry authorities, we captured 25 Bar-headed Geese (8 at Nianhu, 11 at Caohai, and 6 at Napahai) using custom leg snares. This targeted approach involved setting individual snares at foraging areas during early morning hours. Field researchers maintained continuous visual observation of the snares from concealed positions and immediately released any non-target species or approached and manually restrained target geese upon capture. Each captured bird was quickly processed to minimize stress before being fitted with a satellite transmitter. This approach enabled selective capture with minimal disturbance to flocks and maintained a high standard of animal welfare. Each bird was fitted with a satellite transmitter (model Anit-GT0325, Hangzhou Yuehai Technology Co., Hangzhou, China; dimensions: 60 mm × 25 mm × 29 mm; mass: 25 g, approximately 1.7% of body mass). Tagging took place across multiple wintering periods: at Nianhu during the wintering periods of 2014–2015, 2015–2016, 2018–2019, 2019–2020, and 2020–2021; at Caohai during 2017–2018, 2018–2019, and 2019–2020; and at Napahai during 2022–2023 (Supplementary Table S1). A site-by-winter summary of the tagging effort is provided in Supplementary Figure S1 for ease of reference. The wintering period for each tracked goose was behaviorally defined as the duration between its arrival at the wintering wetland (marked by a stabilization of daily movements) and the onset of spring migration, defined as the bird’s final departure from the wintering wetland followed by sustained directional movement. For birds instrumented at the wintering site, the start date denotes the first day of localized movements post-tagging rather than the unobserved true arrival date (see Supplementary Table S1 note). The collected GPS data were cleaned by removing records with clearly erroneous coordinates or elevations, as well as those with Position Dilution of Precision (PDOP) or Horizontal Dilution of Precision (HDOP) ≥ 4 or instantaneous speed ≥ 30 km/h. Diurnal locations were extracted based on local sunrise and sunset times. Due to the significant variation in the number of fixes obtained across the three sites, we subsampled 3000 points from Caohai, 1000 from Nianhu, and 2000 from Napahai as “used” points. To reduce the potential influence of temporal/spatial autocorrelation and unequal sampling among individuals, we adopted this fixed-size subsampling per site and retained only diurnal fixes after rigorous cleaning; together, these steps thinned clustered locations, limited over-representation of particular individuals or periods, and minimized pseudoreplication. We then randomly generated 10,000 available points within each site’s goose activity area, defined by the 95% utilization distribution contour derived from a dynamic Brownian Bridge Movement Model (dBBMM), to construct the random forest habitat selection models. Generating available points within each site’s 95% dBBMM UD constrained the background to empirically accessible space and helped avoid bias from unrealistic availability and spatial clustering.

Based on field surveys, we classified the habitats in each study area into six types: (1) woodland, consisting of large forested areas; (2) grassland, primarily covered by herbaceous vegetation; (3) cropland, used for growing crops; (4) marsh, characterized by low-lying waterlogged terrain, which according to our field surveys typically has a water depth of less than 50 cm; (5) open water, defined as areas of continuous water surface, typically with depths exceeding 50 cm based on our field observations; and (6) built-up land (settlements), comprising areas of buildings and associated paved surfaces. In the habitat selection analysis, we used the minimum distance from goose locations to each habitat type as an indicator of selection preference [38]. Considering the geese’s ecological requirements and the local conditions, we selected nine environmental variables across three categories: nocturnal roost sites, foraging sites, and human-activity covariates. These included distance to nocturnal roost site, distance to cropland, distance to marsh, distance to grassland, distance to open water, distance to woodland, distance to settlement, distance to highway, and distance to rural road. The nocturnal roost sites were identified at the population level for each wetland. Night-time GPS fixes, defined as those recorded between 22:00 and 04:00 local time, from all tracked individuals within a site were pooled and analyzed. We generated a kernel density estimation (KDE) surface using the kernelUD function in the adehabitatHR R package (run under R version 4.2.1), with the smoothing parameter (h) determined by the reference bandwidth method. The core nocturnal roosting area was then objectively defined as the 50% utilization distribution (UD) contour derived from the KDE surface. This approach identified one or two primary, high-use roost locations per site, which corresponded to known communal roosts. In Nianhu, where supplemental feeding sites were present, we also included distance to the supplemental feeding site as an additional variable. The supplemental feeding site is a reserve-managed, human-provisioned grain station situated on a barren islet in the north-western part of the lake where Black-necked Cranes and Bar-headed Geese commonly congregate; we georeferenced its centroid from high-resolution imagery and ground-verified it during the wintering survey. Furthermore, motivated by prior studies reporting associations between Bar-headed Goose foraging distributions and proximity to roads and settlements [39], we included Euclidean distances to the nearest settlement, highway, and rural road as human-activity covariates, treated as coarse proxies of human accessibility. Associations with these variables are interpreted as correlational rather than causal. Specifically, we downloaded Landsat 8 OLI satellite imagery for the study years from the CAS Geospatial Data Cloud (http://www.gscloud.cn (accessed on 20 March 2024). To best represent the land cover during the tracking periods, we selected specific scenes: for the agriculturally stable landscapes of Caohai and Nianhu, single images were sufficient (Caohai: 20 March 2019; Nianhu: 15 January 2019). For Napahai, where water levels fluctuate dramatically, we conducted staged classifications using images from early (20 November 2022), mid- (31 January 2023), and late wintering periods (13 April 2023). The imagery was cropped to the boundaries of each study area in ENVI 5.3, preprocessed, and classified using a supervised classification method. Due to the limited resolution of the Landsat imagery, we used high-resolution Google Earth Pro imagery to assist in selecting training samples [40]. The initial classification result was then refined through a post-processing step to improve its accuracy. This was achieved by visual interpretation against the high-resolution Google Earth baseline: obviously misclassified patches (e.g., shadows misclassified as water, grassland misclassified as marsh) were manually corrected using the editing tools in ArcView 3.3 software. After classification, we assessed map accuracy using confusion matrices derived from an independent validation sample set, which was defined prior to any manual refinement of the classification to ensure an unbiased evaluation. We employed a stratified random sampling design with the six land-cover classes as strata and enforced a minimum of 50 validation samples per class to ensure statistical reliability, particularly for low-prevalence classes. Sample sizes were kept near-balanced across classes, with a mild preferential allocation to locally dominant covers (e.g., cropland and water at Nianhu and Caohai; grassland and woodland at Napahai) to better reflect local landscape composition, maintain comparable chance agreement (Pe) in Kappa calculations, and ensure adequate representation of common misclassifications between ecologically similar types (e.g., grassland and woodland, marsh and water, cropland and built-up land). Since the resolution of Landsat imagery was insufficient to directly identify highways and rural roads, we manually interpreted and digitized major roads and paths in each study area using high-resolution Google Earth Pro imagery. All distance factors were calculated and extracted in ArcGIS 10.8 using neighborhood analysis and nearest-neighbor functions. To accurately capture the dynamic landscape structure resulting from the seasonal hydrological fluctuations at Napahai wetland, we stratified the wintering period of Bar-headed Geese into three distinct phenological stages based on the water recession dynamics: early wintering period (October–November, characterized by post-monsoon high water levels with extensive open water), mid-wintering period (December–January, marked by pronounced water-level drawdown), and late wintering period (February–April, exhibiting the lowest water levels and maximal exposure of foraging habitat). Then we obtained land-use classification results for each period. This staging was specifically adopted to ensure that all distance-based environmental variables were computed against temporally matched land cover, thereby improving the accuracy of the distance factors used in the habitat-selection models.

### 2.3. Data Analysis

The habitat use rate was defined as the proportion of Bar-headed Geese’s location points within each habitat type, quantifying the extent to which each habitat was used by geese. Specifically, we categorized all utilized points by habitat type, counted the number of points in each category, and divided this by the total number of utilized points to calculate the use rate for each habitat type [41]. Data are presented as mean ± standard deviation (SD). Differences in habitat use rates among wetlands were assessed using the Mann–Whitney U test in SPSS (version 27.0).

The dynamic Brownian bridge movement model (dBBMM) is an improved version of the classical Brownian bridge model that provides more accurate estimates of an animal’s activity range [42]. We constructed dBBMMs using the move package in R 4.2.0 and calculated the geese’s utilization distributions (UDs), with the 95% UD contour representing the diurnal activity range. Based on the tracking data error and the size of the activity range, we adjusted parameters in the dBBMM (such as raster, ext, and window size) to generate UD surfaces and extract the corresponding contours. We then used the rgdal package in R 4.2.0 to export the contours as shapefiles and generated available points within each site’s 95% dBBMM UD in ArcGIS 10.8. This constrained the availability of empirically accessible space and helped reduce bias from unrealistic backgrounds and spatial clustering.

The random forest (RF) algorithm is a powerful machine learning method widely employed in ecological studies, owing to its high predictive accuracy, robustness to multicollinearity, and ability to capture complex nonlinear relationships [43,44]. By aggregating predictions across bootstrap-resampled trees, random forests also dampen the influence of spatially clustered observations and emphasize robust population-level patterns—consistent with our study objective. We used the randomForest package in R 4.2.0 to build separate diurnal habitat selection models for Nianhu, Caohai, and Napahai. Model inputs included the actual utilized points of Bar-headed Geese, randomly generated available points within each site’s dBBMM 95% UD contour, and the nine distance-based environmental variables (with an additional “distance to supplemental feeding site” factor for Nianhu). Together with the UD-constrained availability and site-level subsampling of “used” fixes (Section 2.2), this modeling framework helps mitigate over-representation of particular individuals or time periods while keeping inference at the tracked groups level. To improve predictive accuracy, we applied a two-step out-of-bag (OOB) error minimization strategy to tune the key hyperparameters ntree and mtry [45]. We extracted variable importance metrics (based on mean decrease in impurity) from the random forest model and used the rfPermute package (with 1000 permutations of the response variable) to perform permutation tests on each variable’s importance, thereby determining the significance of each predictor’s effect [46]. To visualize the marginal relationships between key predictors and the relative selection probability, we generated partial dependence plots (PDPs) for each site, averaging predictions over the empirical distribution of covariates. These curves allowed us to interpret response directionality and identify potential thresholds, which is instrumental for uncovering nonlinear response patterns critical for understanding behavioral adaptations across heterogeneous landscapes [44].

## 3. Results

### 3.1. Habitat Characteristics

Accuracy assessment using confusion matrices showed that the overall land-use classification accuracy was 95.56% for Nianhu (Kappa = 0.9467), 92.00% for Caohai (Kappa = 0.8994), and an average of 98.27% for Napahai across early, mid, and late wintering periods (Kappa = 0.9792). Although overall classification accuracy is high, characteristic confusion patterns occur in each wetland. At Nianhu, errors predominantly arise in littoral transition zones between open water and marsh, and along field margins between cropland and grassland (Supplementary Table S2). At Caohai, shoreline mosaics result in misclassification of marsh as open water, with occasional confusion between built-up land and cropland occurring near farmyards; limited misclassification of grassland as woodland is also observed in sparse canopy areas (Supplementary Table S3). Across all wintering periods at Napahai (early, mid, and late), confusion between grassland and woodland is associated with canopy-cover gradients, while confusion between marsh and open water occurs in seasonal drawdown zones. Misclassification between cropland and built-up land remains rare and is confined to peripheral areas of rural settlements (Supplementary Tables S4–S6).

**Figure 2:**
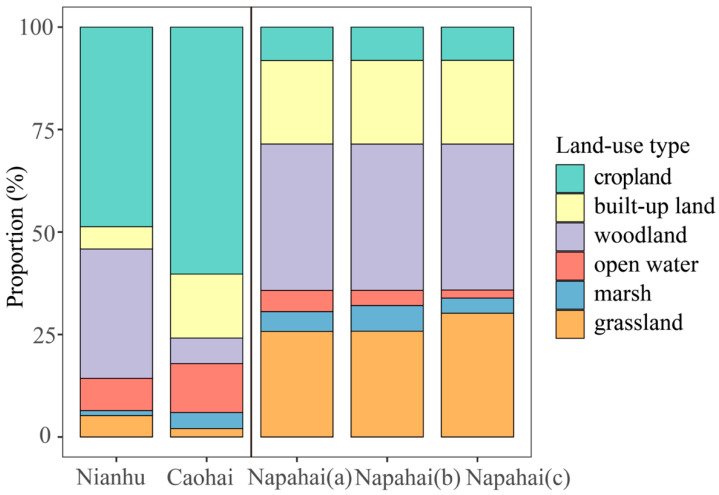
Habitat composition (%) across the three wintering sites. Stacked bars show the proportional area of each land-cover class. From left to right: Nianhu, Caohai, and Napahai in (**a**) early-wintering period, (**b**) mid-wintering period, and (**c**) late-wintering period.

**Figure 3:**
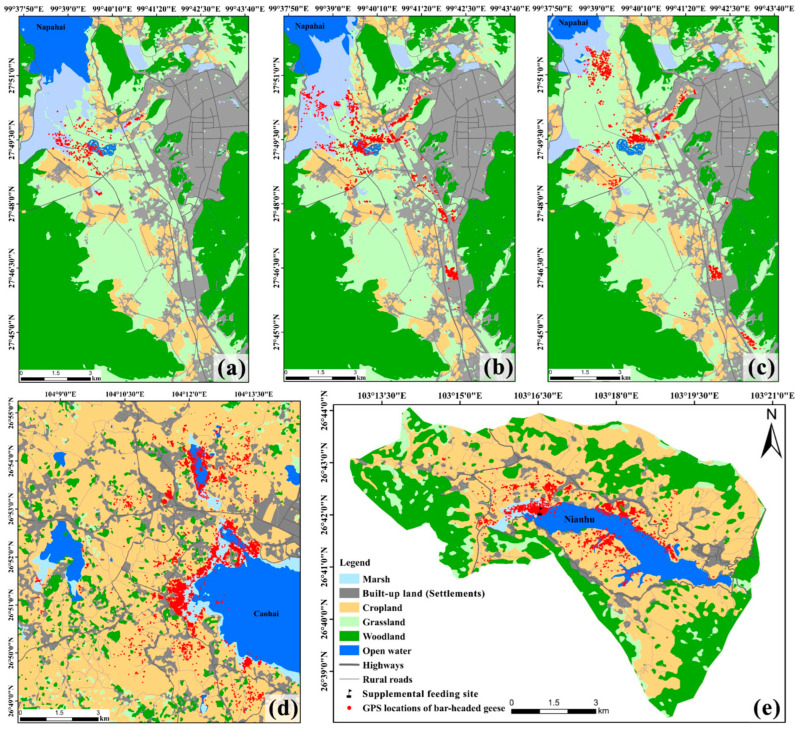
Landscape configuration of the study areas and GPS locations of wintering Bar-headed Geese. (**a**–**c**) Napahai in early, mid, and late winter, respectively; (**d**) Caohai; (**e**) Nianhu. Maps show major land-use classes and GPS locations of Bar-headed Geese. Early wintering period = October–November; mid-wintering period = December–January; late wintering period = February–April. All panels share the same legend and north arrows. Scale bars and map projection (latitude and longitude) are indicated on each panel.

Remote sensing interpretation revealed that the Nianhu study area spans 7295.16 ha, with cropland and woodland dominating the landscape, comprising 49% and 32% of the area, respectively. Open water, built-up land, grassland, and marsh occupy much smaller proportions (approximately 8%, 5%, 5%, and 1%, respectively). Spatially, the eastern part of Nianhu is covered by open water, while the western part is dominated by marsh. Cropland is concentrated along the north and south shores of Nianhu. Built-up land is distributed in bands around the cropland, and woodland occurs along the periphery at higher elevations. Grassland is minimal, appearing only as scattered patches around the cropland and woodland (Table 1; Figure 2 and Figure 3e).

The Caohai study area spans 13,574.48 ha, with a landscape primarily dominated by cropland (approximately 60%), followed by built-up land and open water at approximately 16% and 12%, respectively. Woodland, marsh, and grassland each cover smaller proportions (approximately 6%, 4%, and 2%, respectively). Spatially, open water occupies the center of the Caohai basin, surrounded by marsh. Cropland is distributed across much of the area in clusters, while large patches of built-up land are scattered throughout. Woodland patches are small and fragmented, and grassland is extremely limited, occurring only as a few scattered patches near woodland (Table 1; Figure 2 and Figure 3d).

In the Napahai study area, seasonal fluctuations in water levels cause significant changes in the extent of marsh, grassland, and open water throughout early, mid, and late winter. During early winter, when water levels are high, grassland covers approximately 26% of the area, open water approximately 5%, and marsh approximately 5%. In mid-winter, as water levels drop, grassland remains approximately 26%, open water decreases to 4%, and marsh increases to 6%. By late winter, grassland expands to approximately 30%, open water shrinks to approximately 2%, and marsh declines to 4%. The proportions of woodland, built-up land, and cropland remain relatively stable throughout the winter, at approximately 36%, 20%, and 8%, respectively (Table 2; Figure 2 and Figure 3a–c).

### 3.2. Differences in Diurnal Habitat Use Across Wetlands

**Figure 4:**
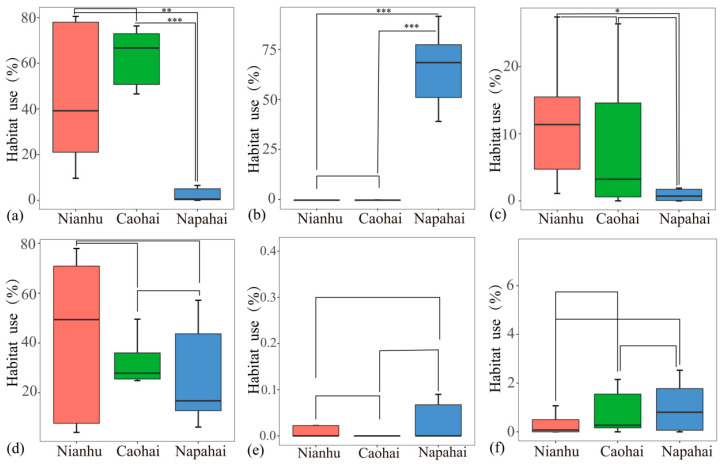
Habitat use (%) of Bar-headed Geese across three wintering sites. Boxplots show the distribution of use rates for six habitat types: (**a**) cropland, (**b**) grassland, (**c**) open water, (**d**) marsh, (**e**) woodland, and (**f**) built-up land. Bold horizontal lines denote median values; boxes span the interquartile range (IQR); whiskers extend to 1.5 × IQR. Asterisks indicate significant pairwise differences between sites (* *p* < 0.05; ** *p* < 0.01; *** *p* < 0.001), as determined by Mann–Whitney U tests.

Bar-headed Geese at Nianhu Wetland primarily utilized cropland (45.88% ± 30.70%) and marsh (42.55% ± 33.17%), followed by open water (11.24% ± 8.75%), with minimal use of other habitat types (<1%). At Caohai Wetland, geese similarly had the highest use of cropland (62.33% ± 12.16%) and marsh (28.61% ± 13.62%), with a lower proportion of open water use (7.65% ± 9.09%) and almost no use of other habitats (<1%). In contrast, at Napahai Wetland, geese primarily utilized grassland (65.92% ± 20.01%), followed by marsh (26.85% ± 21.88%), with lower use of cropland (4.21% ± 7.00%) and minimal use of other habitats (<1%). Statistical tests showed that grassland use was low at both Nianhu and Caohai, with no significant difference between these two sites. In contrast, Napahai geese had a significantly higher grassland use rate than those at Nianhu and Caohai (*p* < 0.001). Cropland use was high at both Nianhu and Caohai, with no significant difference between them, while Napahai geese showed significantly lower cropland use compared to Nianhu (*p* < 0.01) and Caohai (*p* < 0.001). In all three regions, geese used built-up areas at very low rates, with no significant differences observed. Use of open water followed the trend of Nianhu > Caohai > Napahai, with a significant difference in open water use between the Nianhu and Napahai populations (*p* < 0.05). Marsh use did not differ significantly among the three regions (Figure 4).

### 3.3. Differences in Diurnal Habitat Selection Across Wetlands

Building on the observed habitat use patterns described above, we now turn to a more detailed analysis of habitat selection to understand the specific environmental factors influencing the geese’s choices across different sites. The random forest model exhibited high overall accuracy, with OOB error rates of 7.66% for Nianhu, 12.48% for Caohai, and 7.64% for Napahai, indicating strong discriminatory power between used and available points. Analysis of variable importance from these models revealed distinct key drivers of diurnal habitat selection at each wetland.

**Figure 5:**
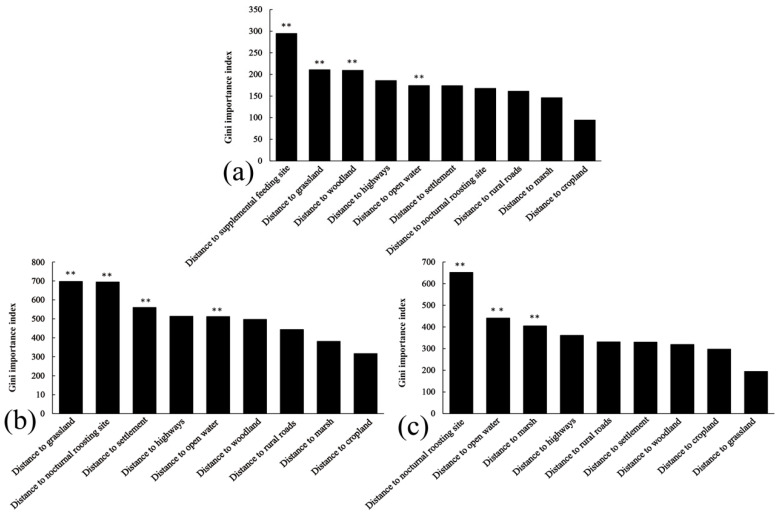
Relative importance of environmental predictors for diurnal habitat selection of wintering Bar-headed Geese, estimated with random-forest models. Bars show the Gini importance index for each predictor; asterisks denote variables with significant permutation importance (** *p* < 0.01; rfPermute). (**a**) Nianhu; (**b**) Caohai; (**c**) Napahai.

**Figure 6:**
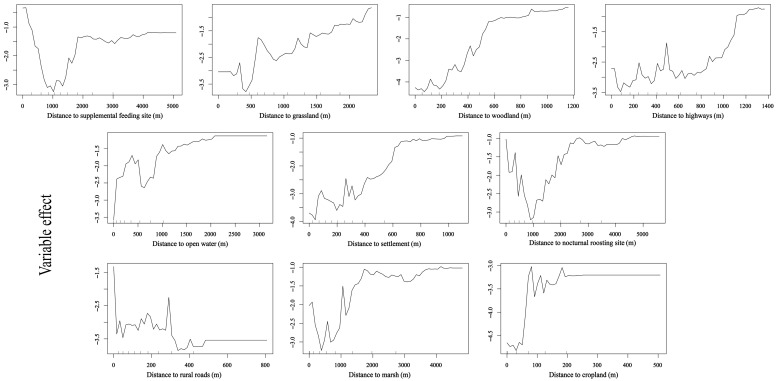
Partial dependence of ten environmental predictors on the relative habitat selection probability by wintering Bar-headed Geese at Nianhu. Curves illustrate the marginal effect of each variable when all other variables are held constant.

The random forest model identified distance to supplemental feeding site, distance to grassland, distance to woodland, and distance to open water as significant factors influencing diurnal habitat selection by geese at Nianhu (*p* < 0.01). At Nianhu, cropland, marsh, and open water were the primary diurnal foraging habitats, whereas woodland and grassland were avoided. Settlements, rural roads, and highways were treated as human-activity covariates. In the Nianhu habitat selection model, the priority of factor categories was as follows: supplemental feeding > avoided habitats > human-activity covariates > nocturnal roost site > main foraging habitat. Specifically, when distance to the supplemental feeding site was zero (i.e., at the supplemental feeding site), relative selection probability was highest, indicating a strong preference for areas near the supplemental feeding site. As the distance from grassland, woodland, and highways increased, relative selection probability also increased, suggesting a preference for areas farther from these features. Within zones of concentrated activity points, the effect of distance to open water on relative selection probability increased and then decreased, with the highest relative selection probability around 400 m. Relative selection probability increased with greater distance from settlements, indicating a preference for areas away from human dwellings. In high-use areas, relative selection probability declined as distance from the nocturnal roost site increased, highlighting the geese’s preference for habitats closer to nocturnal roost sites. The effect of distance to rural roads showed no clear avoidance pattern, as relative selection probability fluctuated in high-use areas. A longer distance to marsh was associated with lower relative selection probability in core use areas, indicating that geese did not strongly prefer locations far from marshes. The effect of distance to cropland increased within the first approximately 100 m, with relative selection probability peaking at around 80 m from cropland (Figure 5 and Figure 6).

**Figure 7:**
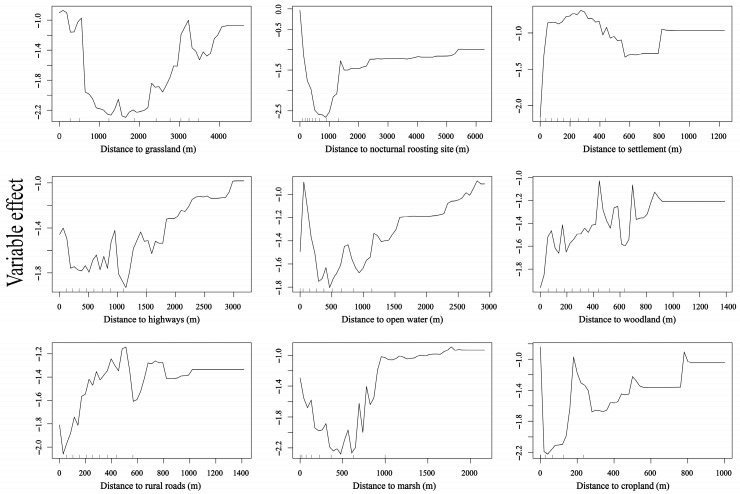
Partial dependence of nine environmental predictors on the relative habitat selection probability by wintering Bar-headed Geese at Caohai. Curves illustrate the marginal effect of each variable when all other variables are held constant.

Distance to grassland, distance to nocturnal roost site, distance to settlement, and distance to open water were significant factors influencing diurnal habitat selection in Caohai (*p* < 0.01). At Caohai, cropland, marsh, and open water were the primary foraging habitats, whereas woodland and grassland were avoided. Settlements, rural roads, and highways were treated as human-activity covariates. In the Caohai habitat selection model, the priority order of factors was as follows: avoided habitats > nocturnal roost site > human-activity covariates > main foraging habitat. The relationship between relative selection probability and distance to grassland was complex: in high-use areas, relative selection probability initially decreased and then increased as distance from grassland grew, indicating avoidance of grassland at most locations. Conversely, relative selection probability dropped sharply with increasing distance from the nocturnal roost site, showing that geese preferred areas close to the roost. As distance from settlements, rural roads, highways, and woodland increased, relative selection probability rose, indicating overall avoidance of these features. In heavily used areas, relative selection probability decreased with greater distance from water, indicating that geese selected habitats closer to the water’s edge. Relative selection probability initially decreased and then increased with distance from marsh, with the lowest probability occurring around 450 m. In heavily used areas, relative selection probability decreased as distance to cropland increased, indicating that geese preferred areas closer to cropland (Figure 5 and Figure 7).

**Figure 8:**
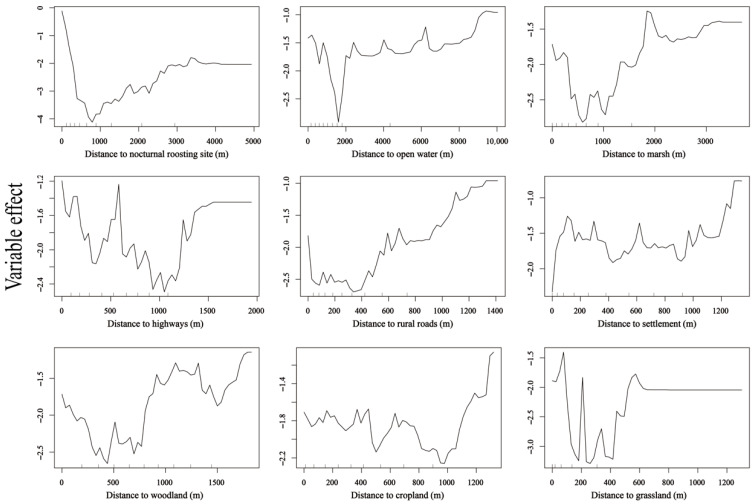
Partial dependence of nine environmental predictors on the relative habitat selection probability by wintering Bar-headed Geese at Napahai. Curves illustrate the marginal effect of each variable when all other variables are held constant.

Distance to nocturnal roost site, distance to open water, and distance to marsh were significant factors influencing diurnal habitat selection in Napahai (*p* < 0.01). At Napahai, grassland, cropland, marsh, and open water were the primary foraging habitats, whereas woodland was avoided. Settlements, rural roads, and highways were treated as human-activity covariates. In Napahai’s habitat selection model, the prioritization of factors was as follows: nocturnal roost site > main foraging habitat > human-activity covariates > avoided habitat. Geese strongly preferred areas near the nocturnal roost site; within zones of concentrated activity points, relative selection probability dropped sharply as distance from the nocturnal roost site increased. In high-use areas, the farther the distance from water and marsh, the lower the relative selection probability, indicating a preference for habitats close to these features. Interestingly, in high-use areas, relative selection probability actually declined with increasing distance from highways and rural roads, suggesting that geese at Napahai did not actively avoid roads. Conversely, relative selection probability increased with distance from settlements, indicating that geese selected locations far from villages. Distance to woodland had a complex effect; beyond ~600 m, relative selection probability increased markedly, indicating a tendency to favor habitats farther from woodlands. Distance to cropland had little effect on selection, suggesting that proximity to cropland did not notably influence habitat choice. Distance to grassland had a significant impact: in high-use areas, relative selection probability dropped rapidly with increasing distance from grassland, indicating a very strong preference for areas close to grassland (Figure 5 and Figure 8).

## 4. Discussion

### 4.1. Habitat Use

Our study revealed clear regional differences in wintering Bar-headed Goose diurnal habitat use patterns across three key wetlands of the Yunnan–Guizhou Plateau. The populations at Caohai and Nianhu primarily utilized cropland habitats (use rates of 45.88% and 62.33%, respectively), while the Napahai population predominantly utilized natural grassland (65.92%). Food resource abundance is a key factor influencing the distribution of wintering Bar-headed Geese [47]. Post-harvest cropland provides abundant high-energy food from leftover crops, attracting geese to forage in fields [22,23,26]. At Nianhu and Caohai, residual crops such as potatoes and oats significantly meet the geese’s energy needs, making cropland their primary foraging habitat under these disturbed conditions. Similarly, studies have shown that grain crops in croplands offer higher nutritional value compared to traditional natural habitats, attracting large numbers of geese [48]. However, this heavy reliance on cropland at Nianhu and Caohai likely reflects an opportunistic adaptation to the scarcity of undisturbed natural foraging areas in these wetlands, rather than an intrinsic preference for cropland.

Notably, Bar-headed Geese can quickly adjust their cropland use in response to local conditions. Liu et al. (2024) found that at Nianhu, when crops in the reserve’s croplands were switched from *Lepidium meyenii* (a crop not eaten by geese) to traditional grains, the geese rapidly shifted their primary foraging habitat from marshes back to croplands, demonstrating their ability to respond to changes in food resources [28].

In contrast, at Napahai, geese mainly utilized seasonal grasslands and marshes formed through natural succession. This is likely because, after the wintering period, water levels drop, large areas of undisturbed, high-quality natural grassland and peat marsh are exposed, providing ample food and space for the geese [49]. Moreover, land-use around Napahai is dominated by ecotourism and pasture, with scattered small croplands of limited crop types, unlike the extensive agricultural areas around Nianhu and Caohai that provide abundant leftover grain. This explains why geese at Napahai make minimal use of cropland.

Furthermore, the observed habitat use shifts in Bar-headed Geese may have implications for interspecific interactions and local biodiversity. As a dominant wintering species, their heavy reliance on croplands in disturbed wetlands could lead to increased competition with other granivorous waterbirds for limited food resources. Conversely, their primary use of natural grasslands in less disturbed areas like Napahai may reduce direct competition and support higher habitat partitioning among species. Future studies should explicitly examine how such habitat-use adaptations influence community structure, resource overlap, and potential facilitation or competition with other waterbird species.

We also found that in all regions, Bar-headed Geese almost never used built-up areas or woodlands, and there were no significant differences among regions in the use of these habitats. This finding aligns with previous research, which has shown that human disturbance is closely linked to waterbird distribution, with waterbirds generally avoiding highly disturbed areas [50]. Densely built-up areas lack the food and secure roosting conditions that geese require, while woodlands have tall, dense vegetation that limits visibility, hindering the geese’s ability to detect danger and forage. Consequently, Bar-headed Geese show extremely low selection for both habitat types.

### 4.2. Habitat Selection

Habitat selection model results indicated that Bar-headed Geese in different regions responded differently to environmental factors in their diurnal habitat selection, reflecting region-specific adaptive behavioral strategies. Geese at Nianhu Wetland showed a strong preference for areas near supplemental feeding sites, a behavior also observed in other waterbirds. For example, Black-necked Cranes wintering in the same reserve congregate around feeding stations [51], and urban Black-headed Gulls (*Chroicocephalus ridibundus*) form large flocks when fed by humans [52]. In the short term, supplemental feeding can improve bird survival during food shortages, but long-term reliance may impair natural foraging abilities and increase the risk of disease transmission [53]. Therefore, the supplemental feeding of wild waterbirds should be managed cautiously.

At Napahai and Caohai, distance to the nocturnal roost site significantly influenced habitat selection, with geese showing a preference for areas near the nocturnal roost site. According to optimal foraging theory, minimizing the distance between diurnal foraging areas and nocturnal roost sites conserves energy, enhancing foraging efficiency and energy storage [54]. Our results align with this theory: in all three wetlands, the relative selection probability decreased as distance from the nocturnal roost site increased.

Additionally, grassland did not serve as a favorable habitat in any of the regions. Although some studies suggest that grassland provides foraging resources for geese [25], at Nianhu and Caohai, grasslands are small, fragmented, and unable to offer sufficient food, causing geese to avoid them.

The influence of woodland on goose habitat selection at Nianhu showed a clear avoidance effect. Woodlands have tall, dense vegetation, which obstructs visibility and hinders the geese’s ability to remain vigilant and forage. This pattern is similar to findings in other species: Common Cranes (*Grus grus*) avoid tall forests when selecting habitat [55], and Crested Ibises (*Nipponia nippon*) prefer open wetlands over forests during the breeding season [56].

Distance to settlements had a significant effect on habitat selection in Caohai, primarily resulting in avoidance behavior. In areas near villages, fallow fields are often plowed or grazed in winter, leaving little crop residue and bare ground. In contrast, croplands farther from villages are less intensively managed, retaining more stubble and spilled grain, which geese can feed on with lower vigilance costs. A recent tracking study of crop damage by geese at Caohai also found that flocks concentrated in hillside fields more than 400 m from villages, foraging briefly during lulls in farming activity before quickly flying off, reflecting their high sensitivity to human disturbance [57].

On the other hand, water-related factors were particularly important for geese at Napahai. The model showed that geese preferred to forage near water, which is consistent with general waterbird ecology: water provides opportunities for drinking, roosting, and refuge from predators [58]. A previous study showed that the activity range of Bar-headed Geese at Napahai is strongly influenced by water area, expanding in the direction of lake recession [59]. Furthermore, Bar-headed Geese tend to select wetlands near rivers or lakes as stopover and wintering sites throughout their life cycle [25]. The dependence of Napahai geese on shoreline areas observed in our study is another manifestation of this pattern.

In summary, Bar-headed Geese tend to select habitats that offer high-energy rewards and are open and safe, reflecting a combination of optimal foraging and risk avoidance strategies.

### 4.3. Behavioral Plasticity

Behavioral plasticity refers to an individual’s ability to flexibly adjust its behavior in response to environmental changes and is a key adaptive strategy for animals [60]. Our study provides empirical evidence that Bar-headed Geese exhibit context-dependent behavioral flexibility across the heterogeneous wetlands. Specifically, the Nianhu wintering population concentrated at supplemental feeding sites and nearby croplands when supplemental feed was abundant; the Caohai population relied heavily on leftover crops in croplands as their primary food source; and at Napahai, where natural habitats are relatively intact, the geese shifted to using expansive natural grasslands and marshes to meet their foraging needs. However, the behavioral shifts we observed represent short-term flexibility rather than evidence of full ecological adaptation to the altered, human-disturbed environments.

The significant regional differences in the use of cropland, grassland, and marsh by geese are also documented in the literature. In the middle of the Yarlung Tsangpo River in Tibet, Bar-headed Geese primarily foraged in cropland (72.1% of their foraging time) [47]. Similarly, at Caohai in Guizhou geese foraged mainly in cropland (73.4%) [26]. In contrast, at stopover sites on the Qinghai–Tibetan Plateau and in the Yellow River Basin, Bar-headed Geese mainly used grassland and open water: 31% (grassland) and 26% (open water) on the Plateau, and 49% (grassland) and 22.5% (open water) in the Basin [25,61]. These findings collectively demonstrate that Bar-headed Geese can flexibly adjust their habitat use strategies to different landscape conditions, exhibiting considerable habitat selection plasticity. Notably, at Nianhu, when crop planting in croplands changed, the geese quickly shifted their main foraging area from cropland to marsh, highlighting the flexibility of their short-term behavioral strategies [28].

Similar plastic behavior is observed in other plateau waterfowl. For example, Black-necked Cranes across various wintering sites in China show highly flexible habitat use, and interventions like supplemental feeding may further alter their foraging preferences, increasing their reliance on artificial food [36]. Other waterbirds also exhibit comparable adaptive strategies: for the critically endangered Siberian Crane (*Grus leucogeranus*) wintering in China, individuals facing natural food scarcity or drought extend their daily foraging time, expand their foraging range, and even alter their diurnal activity patterns to obtain sufficient energy [12]. Similarly, long-term monitoring of multiple waterbird species has shown that in abnormally dry years, many species adjust their migratory stopover site selection and behaviors to avoid areas where water sources have sharply declined [13]. Moreover, some waterbirds facing fragmented breeding habitats or increased human disturbance have changed their breeding timing or migration routes to cope with new environmental pressures [62]. These examples underscore that behavioral plasticity is a crucial strategy for migratory waterbirds to respond to environmental changes. At the population level, such flexibility helps to extend their ecological niche and adaptive range, thereby increasing their tolerance to environmental heterogeneity [63]. For Bar-headed Geese, behavioral plasticity enables individuals to maintain energy intake and survive in the short term; this flexibility may contribute to short-term persistence in certain heterogeneous habitats.

### 4.4. Regionalized Conservation Management

Based on our findings, we recommend implementing conservation strategies tailored to the distinct habitat selection and utilization patterns observed at each wetland, ensuring that management actions are directly informed by the site-specific needs of Bar-headed Geese. At Nianhu Wetland, supplemental feeding exerts a strong attraction for wintering Bar-headed Geese, particularly during extreme weather events or natural food shortages, when it provides additional food resources. However, excessive reliance on supplemental feeding may disrupt the geese’s natural foraging behavior and impair their ability to forage independently. Long-term dependence could even lead to an “ecological trap” [53]. Although such interventions can temporarily alleviate food shortages, they may also increase the risk of disease spread (e.g., avian influenza) by causing birds to congregate and come into more frequent contact with each other [64]. Therefore, we advise that supplemental feeding be strictly regulated: it should be activated only under severe and persistent natural food deficits, restricted spatially to existing provision sites, and limited quantitatively to supplementary amounts. Feeding must be phased out as soon as natural food resources recover. Management decisions should be developed through participatory workshops involving local communities, reserve managers, and agricultural stakeholders to ensure that protocols are ecologically and socio-politically sustainable.

In agricultural wetlands such as Nianhu and Caohai, where croplands constitute a primary foraging habitat (supporting >45% of diurnal use), future land-use planning must integrate the habitat requirements of wintering waterbirds. Our results demonstrate heavy reliance on these human-modified landscapes, underscoring the need to retain crop stubble post-harvest and adopt crop–wetland rotation systems that ensure continuous food availability. Furthermore, landscape-level planning is essential to maintain functional connectivity between roosting and foraging habitats. Conservation strategies should emphasize habitat mosaics, enhance corridor functionality, mitigate edge effects, and incorporate waterbird needs into regional land-use policies to improve landscape permeability and support metapopulation stability across the plateau.

At Napahai, grasslands and marshes are the most important habitats for wintering Bar-headed Geese. These habitats not only provide abundant food resources but also serve as nocturnal roost sites and water sources. Therefore, the protection and management of the grassland ecosystem should be enhanced. Measures such as controlled grazing could help maintain the ecological balance of the grasslands. Additionally, water levels directly affect the geese’s activity range [59]; therefore, they should be regulated according to seasonal hydrological changes to avoid extreme events that could degrade goose habitat. This will ensure that waterbirds can successfully overwinter in a safe wetland environment with sufficient resources. In particular, we recommend that conservation managers delineate core protection zones within existing grassland–marsh mosaics; these zones should be integrated with regional ecological redline policies and natural resource planning frameworks. Furthermore, future land-use decisions should avoid conversion of these critical habitats and consider establishing ecological compensation mechanisms to align local livelihoods with habitat protection goals.

### 4.5. Limitations and Future Directions

Although our study revealed regional differences in the diurnal habitat selection and use of Bar-headed Geese, several limitations merit consideration. First, while we focused on a set of key environmental predictors, other unmeasured factors (e.g., predation risk, vegetation nutritional quality, and climatic variables) could also influence the geese’s behavioral decisions. Their exclusion means we cannot isolate their potential effects, highlighting the need for future studies to incorporate finer-grained environmental data and experimental approaches to uncover the underlying mechanisms. Moreover, detailed analyses of intra-seasonal shifts in habitat use, particularly in relation to agricultural practices and disturbance regimes, would be valuable to complement our results and provide deeper insights into temporal dynamics.

Second, inference in this study is constrained by the uneven distribution of tagged individuals across sites and wintering periods, with small samples in some site–year combinations (Supplementary Figure S1). This imbalance, compounded by the large number of locations per individual, may exacerbate spatial autocorrelation and pseudoreplication. Although we aimed to mitigate these issues through subsampling, defining availability within site-specific dBBMM 95% utilization distributions, and fitting site-level random-forest models to recover population-level patterns, the lack of individual random effects means that some within-individual dependence may remain. Accordingly, our results should primarily be viewed as revealing strong population-level selection patterns for the tracked groups within each wetland; however, inferences for specific wintering periods with small sample sizes should be interpreted with additional caution. Future work should prioritize increasing and balancing per-site–year sample sizes and adopt modeling frameworks—such as mixed-effects models or step-selection functions—that explicitly capture among-individual heterogeneity and spatial dependence.

Third, the accuracy of our habitat selection models is inherently tied to the precision of the underlying land-cover classifications. Although we achieved high mapping accuracy and conducted manual corrections, any misclassification errors would propagate into our distance-based variables, potentially influencing the perceived importance of key drivers. Thus, our inferences are contingent upon the best available remote-sensing data under the current methodology.

Finally, our study quantified habitat use but did not employ availability-standardized metrics, such as electivity indices. Therefore, we cannot definitively determine whether the high use of croplands at Nianhu and Caohai represents an active preference or simply reflects opportunistic use of the most abundant habitat type. We recommend that future studies formally quantify habitat availability to better disentangle true preference from opportunism.

## 5. Conclusions

In conclusion, Bar-headed Geese exhibit region-specific patterns of diurnal habitat use during the wintering period across the Yunnan–Guizhou Plateau’s heterogeneous wetlands. Our comparative analysis showed that the geese in the agriculture-dominated landscapes of Nianhu and Caohai rely heavily on cropland habitats, whereas those in the more natural wetland landscape of Napahai primarily use natural grasslands and marshes. Each population’s habitat selection was driven by a distinct suite of environmental factors, reflecting the species’ behavioral flexibility and ability to adapt to local conditions in the short term. These findings highlight the need for tailored conservation measures in each region. Management interventions should be site-specific: for example, stricter control of supplemental feeding at Nianhu, integration of waterbird habitat requirements into agricultural practices at Caohai, and stronger protection of natural grassland and marsh habitats at Napahai.

## Figures and Tables

**Table 1:** Area (ha) and proportion (%) of habitat types in the Nianhu and Caohai wetlands.

Habitat Type	Nianhu		Caohai	
	Area (ha)	Proportion (%)	Area (ha)	Proportion (%)
Cropland	3553.15	49	8181.07	60
Woodland	2304.06	32	846.64	6
Grassland	379.83	5	277.85	2
Marsh	88.29	1	533.07	4
Open water	573.85	8	1618.84	12
Built-up land	395.98	5	2117.01	16

**Table 2:** Area (ha) and proportion (%) of habitat types at Napahai across early-, mid-, and late-wintering periods.

Habitat Type	Early Wintering Period		Mid Wintering Period		Late Wintering Period	
	Area (ha)	Proportion (%)	Area (ha)	Proportion (%)	Area (ha)	Proportion (%)
Cropland	1352.36	8	1348.11	8	1343.38	8
Woodland	5913.54	36	5910.66	36	5899.51	36
Grassland	4264.05	26	4273.01	26	4998.63	30
Marsh	801.68	5	1038.69	6	616.88	4
Open water	857.31	5	612.46	4	321.01	2
Built-up land	3371.67	20	3377.87	20	3381.39	20

## Data Availability

Data supporting this study will be provided by the corresponding author pending scientific justification and compliance with institutional regulations.

## Supplementary Materials

The following supporting information can be downloaded at: https://www.mdpi.com/article/10.3390/ani15192826/s1, Supplementary Figure S1: Number of satellite-tracked Bar-headed Geese per wintering period (From October to April of the Following Year) at the three study wetlands (Nianhu, Caohai, Napahai), 2014–2022, Supplementary Table S1: Individual satellite-tracking periods and wintering-season dates and duration for Bar-headed Geese at the three study wetlands (Nianhu, Caohai, Napahai), Supplementary Table S2: Confusion matrix for Nianhu (six classes), Supplementary Table S3: Confusion matrix for Caohai (six classes), Supplementary Table S4: Confusion matrix for Napahai-Early wintering period, Supplementary Table S5: Confusion matrix for Napahai-Mid wintering period, Supplementary Table S6: Confusion matrix for Napahai-Late wintering period.

## Abbreviations

The following abbreviations are used in this manuscript:

UD	utilization distribution
dBBMM	dynamic Brownian bridge movement model
