# Supplementary material for: Diurnal Habitat Selection and Use of Wintering Bar-Headed Geese (Anser indicus) Across Heterogeneous Landscapes on the Yunnan–Guizhou Plateau, Southwest China
Source: Animals (Basel). 2025 Sep 28;15(19):2826. doi: 10.3390/ani15192826 (PMC12523391; doi:10.3390/ani15192826)
Supplement: Supplementary file 1 [file animals-15-02826-s001.zip › animals-3830915-supplementary.pdf]

Supplementary Table S1. Individual satellite-tracking periods and wintering-season dates and duration for Bar-headed Geese at the three study wetlands (Nianhu, Caohai, Napahai). Note: For individuals instrumented at the wintering site, the start date denotes the first day of localized movements post-tagging, not the unobserved true autumn arrival date. Duration is computed as (end date − start date + 1) days.

Region	Individual ID	Wintering Periods	Overwinter days
Nianhu	A1	2015.2.1--2015.3.9	37
Nianhu	A2	2015.3.10--2015.4.9	31
Nianhu	A3	2016.3.17--2016.5.1	46
Nianhu	A4	2016.1.23--2016.3.24	62
Nianhu	A5	2019.1.16--2019.3.3	47
Nianhu	A6	2019.12.2--2020.2.29	90
Nianhu	A6	2021.1.12--2021.2.23	43
Nianhu	A7	2018.12.26--2019.1.14	20
Nianhu	A8	2020.12.17--2021.2.23	69
Caohai	B1	2017.11.12--2018.3.8	117
Caohai	B1	2018.11.8--2019.2.28	113
Caohai	B1	2019.12.13--2020.3.5	84
Caohai	B2	2018.1.14--2018.3.9	55
Caohai	B3	2018.11.8--2019.1.13	67
Caohai	B4	2019.11.8--2020.3.5	119
Caohai	B4	2018.11.8--2019.3.13	126
Caohai	B5	2018.12.22--2019.2.28	69
Caohai	B6	2018.12.22--2019.3.3	72
Caohai	B7	2018.12.22--2019.3.3	72
Caohai	B8	2018.12.22--2019.3.2	71
Caohai	B9	2019.1.8--2019.3.10	62
Caohai	B10	2018.12.24--2019.3.3	70
Caohai	B11	2018.12.24--2019.3.3	70
Napahai	C1	2022.12.10--2023.3.23	104
Napahai	C2	2022.12.10--2023.4.5	117
Napahai	C3	2022.12.6--2023.3.31	116
Napahai	C4	2022.11.17--2023.3.28	132
Napahai	C5	2022.10.31--2023.3.30	151
Napahai	C6	2022.11.10--2023.2.3	86

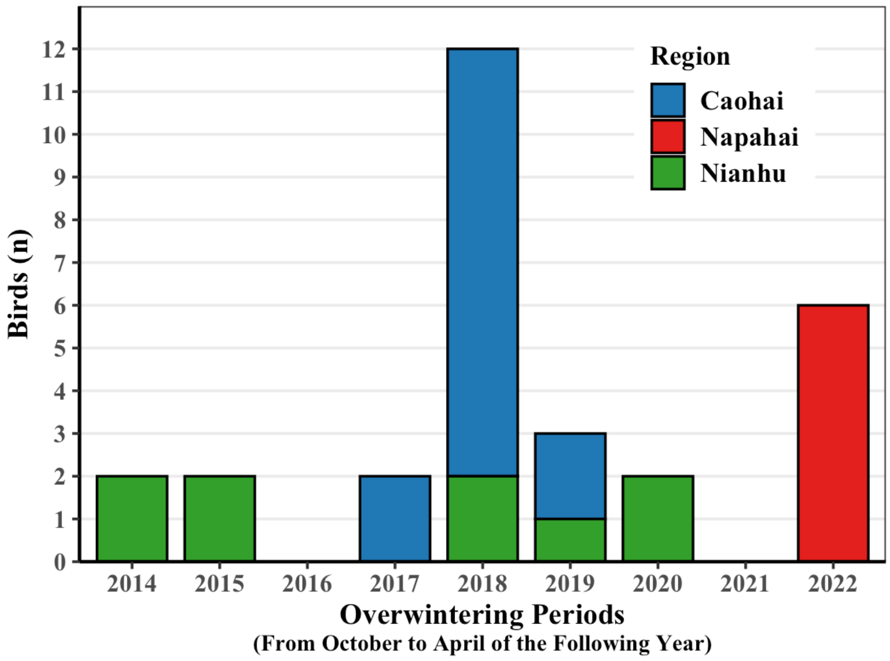

Supplementary Figure S1. Number of satellite-tracked Bar-headed Geese per wintering period (From October to April of the Following Year) at the three study wetlands (Nianhu, Caohai, Napahai), 2014–2022. Bars are stacked by site; colors denote sites (blue = Caohai; red = Napahai; green = Nianhu).

Supplementary Table S2. Confusion matrix for Nianhu (six classes)

Actual \ Predicted	Grassland	Woodland	Cropland	Marsh	Open water	Built-up land	Producer's Accuracy (%)
Grassland	67	0	0	0	0	0	100.00
Woodland	0	72	0	0	0	0	100.00
Cropland	10	0	73	0	0	0	87.95
Marsh	0	0	0	68	0	0	100.00
Open water	0	0	0	10	73	0	87.95
Built-up land	0	0	0	0	0	77	100.00
User's Accuracy (%)	87.01	100.00	100.00	87.18	100.00	100.00	

Note: Overall Accuracy (OA) = 95.56%; Kappa = 0.9467; N = 450

Supplementary Table S**3.** Confusion matrix for Caohai (six classes)

Actual \ Predicted	Grassland	Woodland	Cropland	Marsh	Open water	Built-up land	Producer's Accuracy (%)
**Grassland**	**35**	**7**	**10**	**0**	**0**	**3**	**63.64**
**Woodland**	**0**	**76**	**3**	**0**	**1**	**0**	**95.00**
**Cropland**	**0**	**0**	**170**	**0**	**0**	**0**	**100.00**
**Marsh**	**0**	**0**	**0**	**43**	**12**	**0**	**78.18**
**Open water**	**0**	**0**	**3**	**0**	**127**	**0**	**97.69**
**Built-up land**	**0**	**0**	**9**	**0**	**0**	**101**	**91.82**
**User's Accuracy (%)**	**100.00**	**91.57**	**87.18**	**100.00**	**90.71**	**97.12**	

Note: Overall Accuracy (OA) = 92.00%; Kappa = 0.8994; N = 600

Supplementary Table S4. Confusion matrix for Napahai—Early wintering period

Actual \ Predicted	Grassland	Woodland	Cropland	Marsh	Open water	Built-up land	Producer's Accuracy (%)
Grassland	108	2	0	0	0	0	98.18
Woodland	2	108	0	0	0	0	98.18
Cropland	0	0	90	0	0	0	100.00
Marsh	0	0	0	68	2	0	97.14
Open water	0	0	0	2	73	0	97.33
Built-up land	0	0	1	0	0	84	98.82
User's Accuracy (%)	98.18	98.18	98.90	97.14	97.33	100.00	

Note: Overall Accuracy (OA) = 98.33%; Kappa = 0.9799; N = 540

Supplementary Table S5. Confusion matrix for Napahai—Mid wintering period

Actual \ Predicted	Grassland	Woodland	Cropland	Marsh	Open water	Built-up land	Producer's Accuracy (%)
Grassland	98	2	0	0	0	0	98.00
Woodland	2	118	0	0	0	0	98.33
Cropland	0	0	90	0	0	0	100.00
Marsh	0	0	0	68	2	0	97.14
Open water	0	0	0	2	73	0	97.33
Built-up land	0	0	1	0	0	84	98.82
User's Accuracy (%)	98.00	98.33	98.90	97.14	97.33	100.00	

Note: Overall Accuracy (OA) = 98.33%; Kappa = 0.9799; N = 540

Supplementary Table S6. Confusion matrix for Napahai—Late wintering period

Actual \ Predicted	Grassland	Woodland	Cropland	Marsh	Open water	Built-up land	Producer's Accuracy (%)
Grassland	101	2	0	0	0	0	98.06
Woodland	1	98	1	0	0	0	98.00
Cropland	0	0	89	0	0	1	98.89
Marsh	0	0	0	78	2	0	97.50
Open water	0	0	0	2	88	0	97.78
Built-up land	0	0	1	0	0	76	98.70
User's Accuracy (%)	99.02	98.00	97.80	97.50	97.78	98.70	

Note: Overall Accuracy (OA) = 98.15%; Kappa = 0.9777; N = 540
